# Solar photovoltaic module detection using laboratory and airborne imaging spectroscopy data

**DOI:** 10.1016/j.rse.2021.112692

**Published:** 2021-12-01

**Authors:** Chaonan Ji, Martin Bachmann, Thomas Esch, Hannes Feilhauer, Uta Heiden, Wieke Heldens, Andreas Hueni, Tobia Lakes, Annekatrin Metz-Marconcini, Marion Schroedter-Homscheidt, Susanne Weyand, Julian Zeidler

**Affiliations:** a German Aerospace Center, German Remote Sensing Data Center, Wessling, Germany; b Geography Department, Humboldt Universität zu Berlin, Berlin, Germany; c Remote Sensing Centre for Earth System Research, Universität Leipzig, Leipzig, Germany; d Department of Remote Sensing, Helmholtz-Centre for Environmental Research, Leipzig, Germany; e German Aerospace Center, Remote Sensing Technology Institute, Wessling, Germany; f Remote Sensing Laboratories, University of Zurich, Zurich, Switzerland; g Integrative Research Institute on Transformations of Human-Environment Systems, Humboldt Universität zu Berlin, Berlin, Germany; h German Aerospace Center, Institute of Networked Energy Systems, Oldenburg, Germany

**Keywords:** Mapping, Hydrocarbon spectral index, Urban environment, Renewable energy, Hyperspectral remote sensing

## Abstract

Over the past decades, solar panels have been widely used to harvest solar energy owing to the decreased cost of silicon-based photovoltaic (PV) modules, and therefore it is essential to remotely map and monitor the presence of solar PV modules. Many studies have explored on PV module detection based on color aerial photography and manual photo interpretation. Imaging spectroscopy data are capable of providing detailed spectral information to identify the spectral features of PV, and thus potentially become a promising resource for automated and operational PV detection. However, PV detection with imaging spectroscopy data must cope with the vast spectral diversity of surface materials, which is commonly divided into spectral intra-class variability and inter-class similarity. We have developed an approach to detect PV modules based on their physical absorption and reflection characteristics using airborne imaging spectroscopy data. A large database was implemented for training and validating the approach, including spectra-goniometric measurements of PV modules and other materials, a HyMap image spectral library containing 31 materials with 5627 spectra, and HySpex imaging spectroscopy data sets covering Oldenburg, Germany. By normalizing the widely used Hydrocarbon Index (HI), we solved the intra-class variability caused by different detection angles, and validated it against the spectra-goniometric measurements. Knowing that PV modules are composed of materials with different transparencies, we used a group of spectral indices and investigated their interdependencies for PV detection with implementing the image spectral library. Finally, six well-trained spectral indices were applied to HySpex data acquired in Oldenburg, Germany, yielding an overall PV map. Four subsets were selected for validation and achieved overall accuracies, producer's accuracies and user's accuracies, respectively. This physics-based approach was validated against a large database collected from multiple platforms (laboratory measurements, airborne imaging spectroscopy data), thus providing a robust, transferable and applicable way to detect PV modules using imaging spectroscopy data. We aim to create greater awareness of the potential importance and applicability of airborne and spaceborne imaging spectroscopy data for PV modules identification.

## Highlights

• An approach for photovoltaic modules detection using Imaging spectroscopy data.
• Design and employment of an extensive and scientific dataset upfront.
• It solved the intraclass variability caused by different detection angles.
• Six spectral indices were developed and yielded a map of photovoltaic modules.
• This physics-based approach is robust, transferable and operational.

## 1 Introduction

Due to the increasing energy demand (Wolfram et al., 2012, Sorrell, 2015), the need of cutting down greenhouse gas emissions (Zhang et al., 2019) and the ongoing energy transition process with substantial subsidies (Markard, 2018), the number of solar photovoltaic (PV) modules in operation has increased rapidly in recent years (Tao and Yu, 2015, Green, 2019). Several stakeholders such as environmental authorities, grid operators, manufacturing industries or energy system modelers are interested in monitoring PV system locations and areas, but accurate and publicly accessible databases are not available. Furthermore, these databases need continuous and regular updates. Although in-situ data can be collected through field surveys or citizen science projects, they are costly and/or time-consuming.

In this context, Earth Observation (EO) data offer a suitable alternative. EO data can provide the necessary spatial and temporal resolution to monitor PV modules on a large scale. Promising results have been achieved using color aerial imagery (Malof et al., 2016a, Yu et al., 2018, de Hoog et al., 2020). Malof et al. (2016a) investigated an approach based on supervised random forest classification to automatically identify distributed PV arrays using color aerial images with a spatial resolution of 0.3 m × 0.3 m, and achieved 72% precision and 80% recall. Yu et al. (2018) developed DeepSolar, a deep learning framework that analyzed color spaceborne imagery with a spatial resolution of 0.3 m × 0.3 m to identify the locations and sizes of solar PV modules. The resulting precision was 93.1% and recall was 88.5% in residential areas, while precision was 93.7% and recall 90.5% in non-residential areas. Leveraging its high accuracy and scalability, they constructed a comprehensive high-fidelity solar deployment database for the US. However, identifying solar PV modules across large regions remains challenging due to the requirement of high-resolution (typically 0.3 m/pixel or finer) imagery, difficult identification of solar PV modules in many situations (such as dark PV modules on dark roofs), and confusion of many other types of structures (such as solar hot water systems, roads, and even pools) to PV modules (de Hoog et al., 2020). This is because PV modules are composed of materials that typically include fully transparent glass covers for protection, highly transparent Ethylene Vinyl Acetate (EVA) films, and the core PV cell. In addition to these reasons, these methods require large, elaborated and pixel-accurate labeled data sets for training and validation (Malof et al., 2016a, Malof et al., 2016b, Yuan et al., 2016, Camilo et al., 2018).

Instead of providing only RGB broad band spectra as color aerial imagery, imaging spectroscopy data can generally improve the separability of surface materials since its near continuous spectral information, with hundreds of narrow spectral bands can map the material-specific absorption characteristics (Herold et al., 2004, Heiden et al., 2007). Thus, more detailed spectral properties of PV modules can be derived from imaging spectroscopy data. So far, very few studies focus on PV detection with imaging spectroscopy data. Czirjak (2017) showed that PV modules have a unique spectral signature that is consistent across multiple manufacturers and construction methods and is therefore detectable in imaging spectroscopy data, i.e., using an adaptive cosine estimator to detect PV modules. In addition, Czirjak (2017) developed the Normalized Solar Panel Index (NSPI) to mitigate false positives by eliminating pixels that do not exhibit key spectral features of the reflectance spectrum of PV panels. The NSPI is designed to detect the steep increase in reflectance that typically occurs in spectral signatures of solar PV modules around 1.00 μm. Karoui et al. (2019) attempted to use Non-negative Matrix Factorization (NMF) algorithms to apply Linear Spectral Unmixing (LSU) on imaging spectroscopy data for PV detection. Their study concludes that the proposed approaches (Grd-Part-NMF and Multi-Part-NMF) are superior to the previous ones (Grd-NMF and Multi-NMF), which is a promising progress. However, it is important to note that the previous NMF approaches (Grd-NMF and Multi-NMF) are not PV detection approaches. Moreover, only one mean spectrum of the ground-measured PV modules spectra was considered as the known spectrum but different types of PV were not considered, which means a lack of variation of the PV spectra in the training phase. In addition, Karoui et al. (2019) did not consider the spectral variability caused by different inclination or detection angles, which is a limitation of the linear unmixing methods in principle since these methods are generally considered when the landscape of the observed scene is flat and the irradiance is homogeneous (Dobigeon et al., 2013). Furthermore, Karoui et al. (2019) did not consider materials that have similar spectra to PV panels, such as polyethylene materials and oil, which have similar double absorption feature at 1.73 μm due to their hydrocarbon content, and water, which has similar low reflectance in the VNIR region. For these reasons, Karoui et al. (2019) attempted the creative and meaningful experiment on PV detection using imaging spectroscopy data with LSU, but the detection of PV modules were not accurate enough, while a simple one-class classification generally achieved better results.

Therefore, PV modules detection using imaging spectroscopy data should focus on the physical characteristics and the spectral uniqueness of PV modules. PV modules commonly consist of several layers, including fully transparent glass covers for protection, highly transparent EVA films, and the core PV cell. EVA is a hydrocarbon-bearing material, so regardless of how well EVA transmits solar energy, the hydrocarbon absorption exists at 1.73 μm. Crystalline silicon (C-Si), as a common PV cell material, has a strong absorption in the visible (VIS) region, resulting in low reflectance of PV modules in the VIS region. In addition, it has a decreasing absorption between 0.99 μm and 1.15 μm, resulting in a steep reflectance increase in this spectral region. Moreover, like most hydrocarbon surface materials, PV modules have a strong absorption around 2.2 μm. However, PV detection using imaging spectroscopy data must cope with the vast spectral diversity of urban materials and related characteristics, commonly classified as intra-class variability and inter-class similarity. Intra-class variability means the spectral variability within the material class, and inter-class refers to the spectral similarity among different material classes (Zhang et al., 2006, Somers et al., 2011). Intra-class variability can be caused by several factors, such as color, coating, degradation of the material and illumination of the material as well as preprocessing of the acquisition data (Heiden et al., 2007). In PV detection, the spectral variability caused by different tilt angles of PV or detection angles of sensors is common and has therefore attracted our attention. In addition, polyethylene covered open surfaces, roofing polyethylene and synthetic turf on sports fields, which are hydrocarbon-bearing materials similar to EVA in PV modules, could cause the spectral inter-class similarity, and therefore are another problem to be addressed in PV modules detection.

The objective of this study is to detect PV modules using airborne imaging spectroscopy data. Specifically, we aim to address 1) the spectral intra-class variability caused by different viewing and illumination angles, which is always present in PV detection; 2) the spectral inter-class similarity that occurs mainly between PV modules and other hydrocarbon-bearing materials; 3) as well as to apply and validate the developed spectral indices on the city of Oldenburg, Germany. To address these questions, we firstly identify specific spectral features of PV in the optical spectral range and introduce spectral indices based on laboratory spectra-goniometric measurements with different detection angles and a large labeled HyMAP image spectral library. These indices are then applied to airborne HySpex images acquired over Oldenburg, Germany.

## 2 Data and study area

### 2.1 Laboratory spectra-goniometric spectral library

**Fig. 1:**
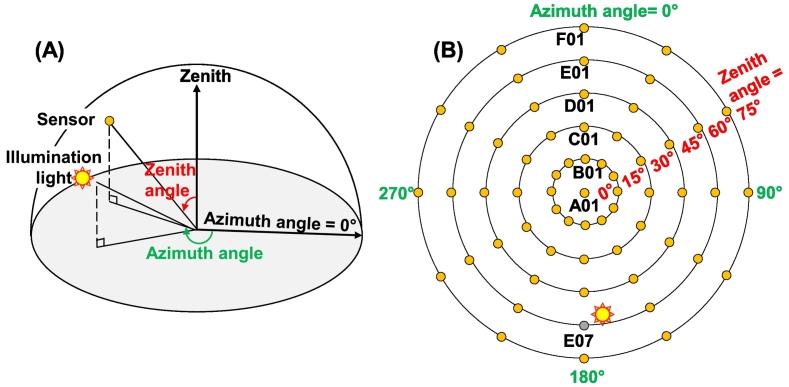
The measurement set-up with an ASD sensor in a goniometer. (A) The material was placed on the gray platform, and measured with a fixed illumination light and a movable ASD sensor. (B) the arrangement of the total 61 measurement positions and their labels. Detection E07 was treated as an abandoned measurement because it was close to illumination light and affected.

Five materials were measured with the ASD spectrometer with a 3° field of view installed on the LAGOS goniometer (Schopfer et al., 2008) (Fig. 1A), including two bitumen materials for roof covers, a monocrystalline PV module, a polycrystalline PV module, and a hydrogen carbonate (PVC) material normally applied on large flat roofs (Table 1). For each material, a total of 61 measurements with different detection angles were collected (Fig. 1B) as well as one measurement with the white reference. Specifically, the 61 measurements cover zenith angles of 0° to 75° with an interval of 15° and azimuth angles of 0° to 330° with an interval of 30°. These measurements were labeled by alphabet for zenith angle (from A to F) and by number for azimuth angle (from 1 to 12) except for zenith angle = 0°. The measurement at E07 (zenith angle is 60°, and azimuth angle is 180°) was excluded, because the illumination source (55° of zenith angle, and 171° of azimuth angle) was between the sample and the optic of the spectrometer. Raw data were recorded as radiance and processed to reflectance by normalizing the radiance with the white reference. Therefore, a total of 60 reflectance spectra were available for each material. Each spectrum covers the spectral range from 350 to 2500 nm, with a spectral resolution of 1 nm.

**Table 1:** Materials and spectra in the laboratory spectra-goniometric measurements and HyMap image spectral library.

Laboratory spectra-goniometric measurements					
Materials	Spectra number	Color	Detail		
Bitumen material A	60	red	age∼2017		
Bitumen material B	60	gray	age∼2017		
PV material A	60	black	monocrystalline PV cell		
PV material B	60	dark blue	polycrystalline PV cell		
PVC	60	black	hydrogen carbonate		

HyMap image spectral library					
Materials	Spectra	Materials	Spectra	Materials	Spectra
Roofing tiles	624	Roofing tar	15	Siliceous sand	31
Roofing concrete	352	Roofing glass	44	Humus soil	96
Aluminum	188	Vegetated roof	108	River	466
Copper	123	Concrete	157	Pond	183
Zinc	159	Asphalt	339	Pool	34
Polyvinyl chloride (PVC)	244	Concrete pavement	10	Coniferous trees	248
Roofing polyethylene	359	Cobblestone	10	Deciduous trees	277
Polyethylene surface	89	Loose chippings	184	Dry vegetation	19
Tartan	22	Railway tracks	65	Meadow	187
Synthetic turf	264	Vegetated railway tracks	28	Lawn	415
Roofing bitumen	287				

### 2.2 HyMap image spectral library

An image spectral library extracted from airborne hyperspectral data was also included in this study. The library was mainly derived from imaging spectroscopy data recorded over Munich, Dresden, Potsdam and Berlin, Germany in 1999, 2000, 2004 and 2007 (Segl et al., 2003, Heiden, 2004, Bochow, 2010, Heldens, 2010). All data were acquired with the HyMap sensor (Cocks et al., 1998). The radiometrically and atmospherically corrected HyMap data have 128 bands, of which three bad bands were removed, which were the first band of the visible (VIS) spectrometer, and the first and second band of the near infrared (NIR) spectrometer (Heldens, 2010). The spectral library was extracted based on the method of Segl et al. (2003), developed by Heiden et al. (2012) and extended by Heldens (2010). It contains 5627 labeled spectra of 31 material classes. Each spectrum in this library has 125 spectral bands ranging from 450 to 2500 nm. The HyMap spectral library includes several polyethylene materials, i.e., roofing polyethylene, polyethylene surface, and synthetic turf, which have a hydrocarbon absorption similar to PV modules. This allowed us to collect the spectral features of these similar materials, and remove them in the PV detection.

### 2.3 HySpex images

**Fig. 2:**
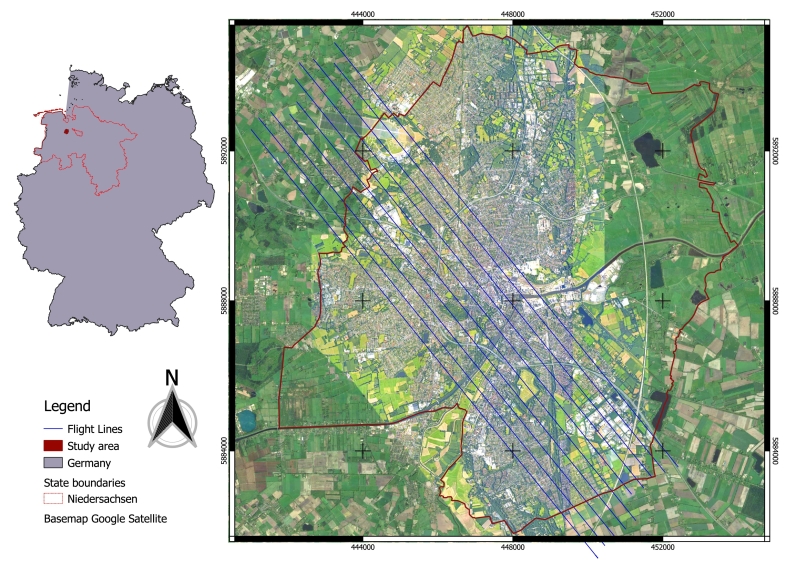
The study area – Oldenburg in the northwest of Germany, covered with ten HySpex images.

Ten imaging spectroscopy data sets were collected from a flight campaign carried out in July 2018 covering Oldenburg with the HySpex sensor (see Fig. 2). The HySpex system has two cameras covering the spectral ranges of visible near infrared (VNIR) and short-wave infrared (SWIR) region. The VNIR sensor records the spectral range from 416 to 992 nm with 160 channels at a spatial resolution of 0.6 m × 0.6 m. The SWIR sensor covers the spectral range from 968 to 2498 nm in 256 channels at a spatial resolution of 1.2 m × 1.2 m. To work with the same reference frame and the whole spectral range, the VNIR and SWIR images were co-registered (Schwind et al., 2014) and resampled to the same spatial resolution of the SWIR sensor, which is 1.2 m × 1.2 m. More details about the characteristics of the HySpex system are provided in Köhler (2016).

HySpex Level 2A data were provided for this campaign. After system correction, the data were ortho-rectified, and the surface reflectance was calculated with the ATCOR4 atmospheric correction software (Richter et al., 2011) for each HySpex flight line. This pre-processing was carried out by OpAIRS of the Remote Sensing Technology Institute (IMF) of German Aerospace Center (DLR), and described in detail in Köhler (2016). It should be noted that the uncertainty in the relative geolocation between two adjacent flight lines can be up to 3 pixels, and that a further systematic/non-systematic displacement to the validation data of 1–2 pixels exists. The effects are described in Section 3.4.

### 2.4 Study area

The city of Oldenburg is located in the northwest of Germany (see Fig. 2), and covers an area of 103 km^2^. The study area captured by the HySpex images includes a variety of building types with different installations of PV modules. A large PV power plant is located on the old airfield in northwestern Oldenburg. Slightly further south are two university campuses, Haarentor and Wechloy, and the Institute for Networked Energy Systems of the DLR. The city center of Oldenburg is dominated by dense perimeter block developments with varying roof materials and few open spaces. The south of Oldenburg is characterized by several industrial areas with halls and warehouses, as well as large areas with many semi-detached and detached houses, some of which are covered with PV modules.

## 3 Methods

**Fig. 3:**
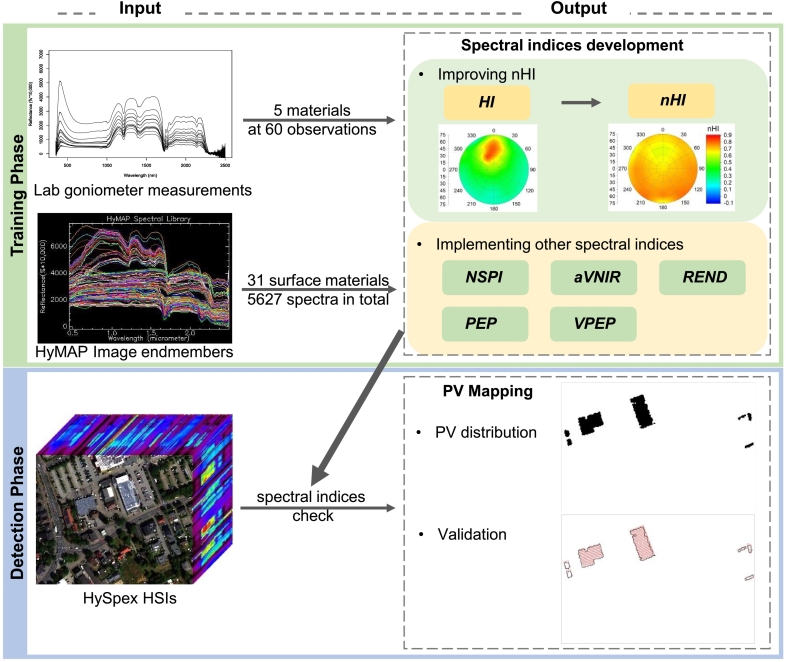
The study workflow.

To capture and describe the spectral characteristics of PV modules, we applied a set of spectral indices using imaging spectroscopy data. The study workflow is shown in Fig. 3.

### 3.1 Hydrocarbon index normalization

**Fig. 4:**
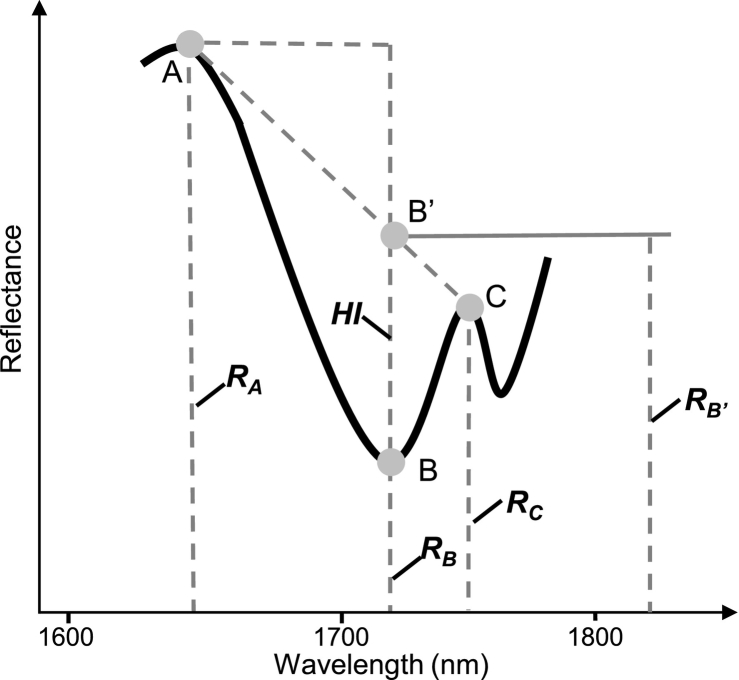
Demonstration of the spectral absorption in the reflectance of hydrocarbon-bearing materials (modified from Kühn et al. (2004)). A, B, and C are the points on the reflectance line, and B’ is a point on the continuum line of points A and C. Accordingly, their reflectance values are *R*_A_, *R*_B_ and *R*_C_, and wavelengths are *λ*_A_, *λ*_B_ and *λ*_C_. The distance between points B and B’ were defined as *HI* (Kühn et al., 2004).

The diagnostic spectral characteristics of hydrocarbon in the SWIR were reported by Cloutis (1989), which revealed the hydrocarbon absorption feature centered near 1.73 μm arising from the various C-H stretching overtones and combination bands. Since then, the basic ability of hyperspectral systems was explored to detect hydrocarbon features in the SWIR. Hörig et al. (2001) realized this capability using airborne HyMap imaging spectroscopy data to map and delineate oil-contaminated soils based on the absorption feature. Based on this, Kühn et al. (2004) proposed the Hydrocarbon Index (HI) (see Fig. 4 and Equation (1)) that measures the depth of the spectral absorption at 1.73 μm to identify the presence of hydrocarbon-bearing material. This HI converts multi-band data into a single band, which is straightforward to use for detecting the presence of hydrocarbon-bearing materials.

(1) $$\mathrm{HI}=R_{B^{\prime }}-R_{B}$$

where

(2) $$R_{B^{\prime }}=(\lambda_{B}-\lambda_{A})\frac{R_{C}-R_{A}}{\lambda_{C}-\lambda_{A}}+R_{A}-R_{B}$$

However, HI does not consider the spectral variation of hydrocarbon-bearing materials, i.e., the spectral intra-class variability of PV modules due to color, coating, degradation of the material and orientation of the material to the sensor etc. (Heiden et al., 2007, Clark and Roush, 1984, Clark et al., 2003, Sahib, 2019). Clark and Roush (1984) proposed a consistent band depth concept to reduce topographic and atmospheric effects by calculating band depth with the support of spectral continuum. By combining the concepts of band depth and HI, we performed a normalization procedure for the HI to minimize the influence of different detection angles. The normalized HI, called nHI, is calculated by dividing the HI by the $R_{B^{\prime }}$ (see Equation (3)).

(3) $$\mathrm{nHI}=\frac{\mathrm{HI}}{R_{B^{\prime }}}=\frac{R_{B^{\prime }}-R_{B}}{R_{B^{\prime }}}$$

The HI was calculated by using 1705 nm, 1729 nm and 1741 nm as points A, B and C in Kühn et al. (2004). Since nHI uses the concept of continuum-removed absorption band and is calculated by dividing the band depth of each channel by the reflectance at the band center, points A and C should be at the spectral shoulders. Therefore, we selected 1669 nm and 1746 nm as the points A and C. The point B is still 1728 nm, which is the center of the hydrocarbon absorption feature. Given the different spectral resolution of sensors used in this study, the exact wavelengths of points A, B and C can be slightly modified and adjusted to suit particular sensors.

### 3.2 Additional spectral indices

**Fig. 5:**
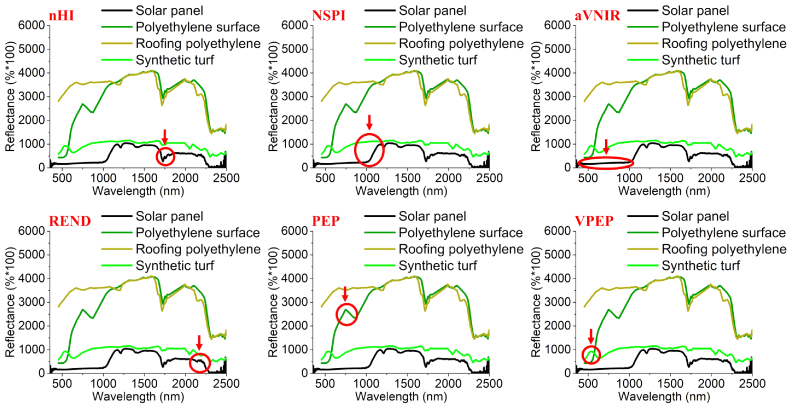
Demonstration of six spectral indices. The PV spectrum is from laboratory spectra-goniometric measurement (monocrystalline PV module, D10). The spectra of polyethylene surface, roofing polyethylene and synthetic turf are from the HyMap image spectral library.

Instances of different material classes may exhibit highly similar spectral features, which is referred to as inter-class similarity. The hydrocarbon absorption feature of PV modules at 1.73 μm is not unique, while other hydrocarbon-bearing materials also exhibit this feature. Therefore, these polyethylene materials such as roofing polyethylene, polyethylene surface and synthetic turf, should be constrained by additional specific indices in case they are misclassified as PV modules. Thus, four additional spectral indices are developed in this study to accurately distinguish PV modules from other hydrocarbon-bearing materials. Including the above-mentioned nHI, a total of six spectral indices based on the physical characteristics and corresponding spectral features of PV modules are shown in Fig. 5, and their equations are in Table 2.

**Table 2:** Spectral indices developed and used in the study.

Details	Expression (wavelength/nm)	Threshold
normalized Hydrocarbon Index (nHI)	(4) $\mathrm{nHI}=\frac{R_{B^{\prime }}-R_{B}}{R_{B^{\prime }}}$	>0.18
Normalized Solar Panel Index (NSPI) (Czirjak, 2017)	(5) ${\mathrm{NSPI}}_{\mathrm{HySpex}}=\frac{R_{1153}-R_{991}}{R_{1153}+R_{991}}$	>0.15
average reflectance in Visible and Near Infrared Range (aVNIR)	(6) $\mathrm{aVNIR}=\mathrm{Mean}\left(\sum_{i=500}^{1000}R_{i}\right)$	<2000
Reflectance drop around 2200 nm (REND)	(7) $R_{2100}>R_{2200}>R_{2300}$	- -
A reflectance peak between 0.6 and 0.8 μm, to differentiate from PolyEthylene surface Peak (PEP)	(8) $\mathrm{PEP}=R_{750}-R_{650}-\frac{10}{11}\left|R_{860}-R_{650}\right|$	<200
A reflectance peak in visible range (VPEP), to differentiate from synthetic turf	(9) $\mathrm{VPEP}=R_{630}-R_{470}-\frac{7}{16}\left|R_{540}-R_{470}\right|$	<200

NSPI exploits the rapid increase in the reflectance spectra of PV modules (see Fig. 5) caused by C-Si absorption. C-Si PV modules include poly-C-Si and mono-C-Si. Both types exhibit similarly decreasing energy absorption capabilities from 600 nm to 1150 nm (Schinke et al., 2015, Deng et al., 2017), resulting in an increase in reflectance. As the market share of silicon solar cells currently exceeds 90% (Silvestre et al., 2018), this feature can be treated as another main spectral feature of PV modules.

The index of average reflectance in the VNIR (aVNIR) (see Fig. 5) targets strong spectral absorption features in the VNIR region of PV materials. Common and traditional PV modules have a low average reflectance between 500 nm and 1000 nm (Czirjak, 2017), which is used to absorb more solar energy in this spectral range. For example, monocrystalline PV cells are blackish, and polycrystalline PV cells are dark bluish. In this study, we specified an experimental threshold for aVNIR (see Table 2) to primarily eliminate roofing polyethylene.

The index of reflectance drop around 2200 nm (REND) (see Fig. 5 and see Equation (7) in Table 2) addresses the typical hydrocarbon absorption properties, since the spectral region from 2200 to 2500 nm is affected by numerous overlapping combination and overtone bands. The sheer number of overtone bands causes reflectance to decrease substantially around this region (Herold and Roberts, 2005). These overtone bands can be assigned to the *CH*_2_ and *CH*_3_ stretch and bend, carbonyl-carboxyl C-O stretch, and aromatic carbon stretch (Cloutis, 1989).

The aVNIR and REND (see Fig. 5) are physically meaningful spectral indices, but cannot constrain polyethylene surface and synthetic turf misclassified as PV modules. For this reason, the PolyEthylene Peak (PEP) index and the PolyEthylene Peak in Visible range (VPEP) were proposed explicitly for polyethylene surface and synthetic turf, respectively. The PEP feature of polyethylene surface is caused by a strong spectral absorption of polyethylene surface at 800 nm to 900 nm and results in a reflectance peak between 650 nm and 860 nm. The VPEP feature is due to the fact that synthetic turfs often have visual colors, such as green artificial playgrounds and red artificial running tracks.

### 3.3 PV mapping on Oldenburg

The ten HySpex imaging spectroscopy flight lines were converted from uncompressed band sequential (BSQ) binary files to LZW compressed interleaved geoTIFF, which reduced the data size by a factor of 15 and allowed the data to be read efficiently in chunks for processing. Six spectral indices were sequentially applied to the geoTIFFs, and the pixels that could pass criteria of all spectral indices were treated as PV module covered pixels (see Table 2). Some classification errors occurred at class boundaries due to spectral mixing within a pixel. These misclassified areas are small compared to the correctly classified areas. Within a class, there are anomalous pixels due to noise in the data. These areas are small compared to the overall pattern. Since one pixel of PV modules is rare at a spatial resolution of 1.2 m × 1.2 m of airborne imagery, it was considered as a noisy pixel. Therefore, we applied morphological filtering (clump classes) to remove these noisy pixels and maintain the border pixels.

### 3.4 Validation

Four subsets were selected to evaluate the accuracy of PV detection, as shown in Fig. 10. Each subset covered 301.2 m × 199.2 m (251 × 166 pixels) in size and was co-registered to the HySpex data. Subset A is dominated by a PV power plant, subset B is the area where campus and institute are located, subset C is a residential area, and subset D covers an industrial area. We manually collected validation data on these four subgroups on airborne 3K photos in combination with field checks. The 3K photos were collected at a similar time as the HySpex data, and have a spatial resolution of 10 cm. Due to an uncertainty in geolocation between the 3K photos and the HySpex data sets, a manual shift of up to one pixel (1.2 m) was applied to the PV mapping images to better match the validation data. The Overall Accuracy (OA), Producer's Accuracy (PA) and User Accuracy (UA) were obtained with a pixel-to-pixel comparison using the confusion matrix, while treating PV and non-PV as two classes.

## 4 Results

### 4.1 Dealing with the spectral intra-class variability

**Fig. 6:**
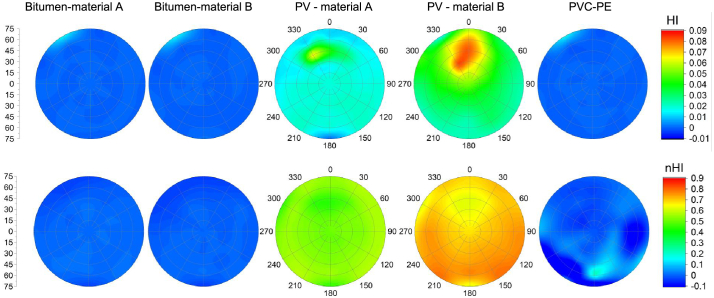
The polar plots of HI and nHI for bitumen material A, bitumen material B, PV material A, PV material B, and PVC with different detection positions. The HI and nHI values were interpolated.

The HI and nHI values of the five materials at different detection angles were calculated, interpolated, and shown in Fig. 6. Bitumen material A, B and PVC material show a value close to zero, because the absence of a distinct hydrocarbon absorption feature at 1.73 μm, and therefore result in HI and nHI values close to zero. Two PV materials have higher HI and nHI values from their hydrocarbon features. For HI values, two PV materials show variation among different detection angles. The closer the angle of reflection directly opposite to the incident light (zenith angle 55°, and azimuth angle 171°), the higher the HI values are acquired. However, their nHI values show a greatly minimized variation among the detection angles.

**Fig. 7:**
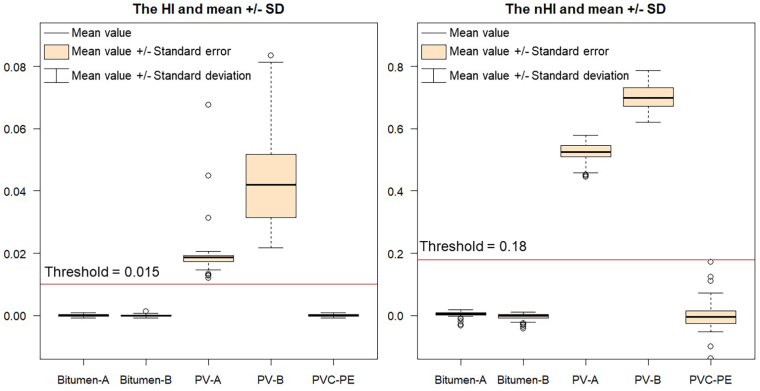
The comparison of HI and nHI with their mean values, ± standard deviations, ± standard errors and outliers.

To statistically compare HI and nHI, we show that both HI and nHI can separate PV materials from three other materials of the spectra-goniometric data set, and we can define threshold values of 0.015 and 0.18 for HI and nHI respectively, as depicted by Fig. 7. However, nHI values show greater separability between PV materials and other materials. In addition, Fig. 7 also shows that HI values of the two PV materials have larger variation among different detection angles than nHI values of PV materials, which are relatively stable.

**Fig. 8:**
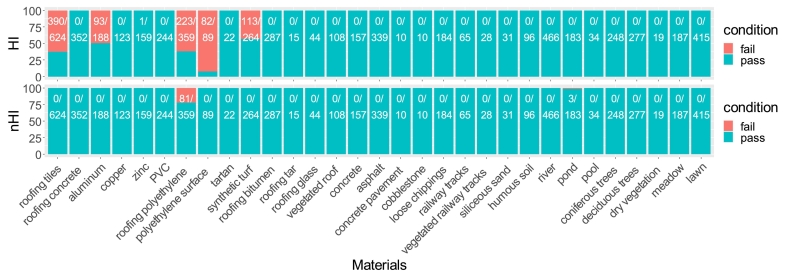
The HI and nHI check of 31 materials in HyMap image spectral library. The ratio between the number of passed spectra and the number of total spectra for each specific material is also displayed.

The HyMap image spectral library was also used to calculate the HI and nHI values (see Fig. 8). For the HI values, the spectra of half of the tartan, half of the roofing polyethylene, a few roofing aluminum, roofing concrete as well as some pond spectra failed the HI threshold check, meaning that their values were above the thresholds and assumed to be PV. This would cause difficulties in the following PV detection, since these materials could be recognized as PV by HI check. As for nHI, some roofing polyethylene spectra have a strong absorption at 1.73 μm, and therefore failed nHI threshold check. In addition, some pond spectra also failed this nHI check. Therefore, although nHI has better performance as it removes the intra-class variability due to different detection angles, it is not good enough to independently distinguish PV from the other surface materials, and some more spectral indices are required to constrain the hydrocarbon-bearing materials.

### 4.2 Dealing with the spectral inter-class similarity

**Fig. 9:**
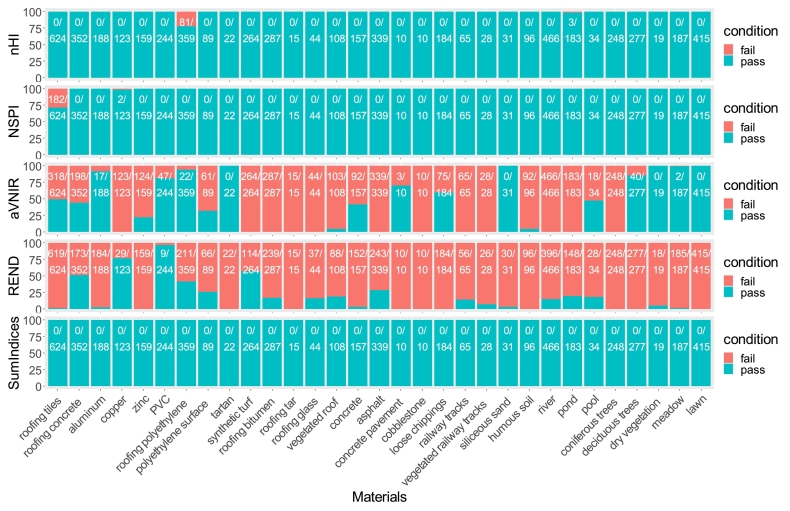
The independent check of four spectral indices (nHI, NSPI, aVNIR, REND) and their combined check (SumIndices) with HyMap image spectral library. The number of failed spectra/number of overall spectra for each specific material is also shown.

NSPI, nHI, aVNIR, REND were independently applied on the HyMap spectra library, as they can specify different materials based on typical PV features (see Fig. 9). NSPI performed quite well, only some spectra of roofing tiles and a few copper spectra failed in this check. The aVNIR had a quite good performance with checking tartan (0 fail), most roofing polyethylene (22/359 fail proportion). REND index independently did not perform well on this check, because the addressed feature is present in most materials. We still keep REND to eliminate false positives caused by image noise. The Sumindices gave sufficient results for this large spectral library and no spectral fail occurred with the combination of these four indices.

### 4.3 PV mapping result

**Fig. 10:**
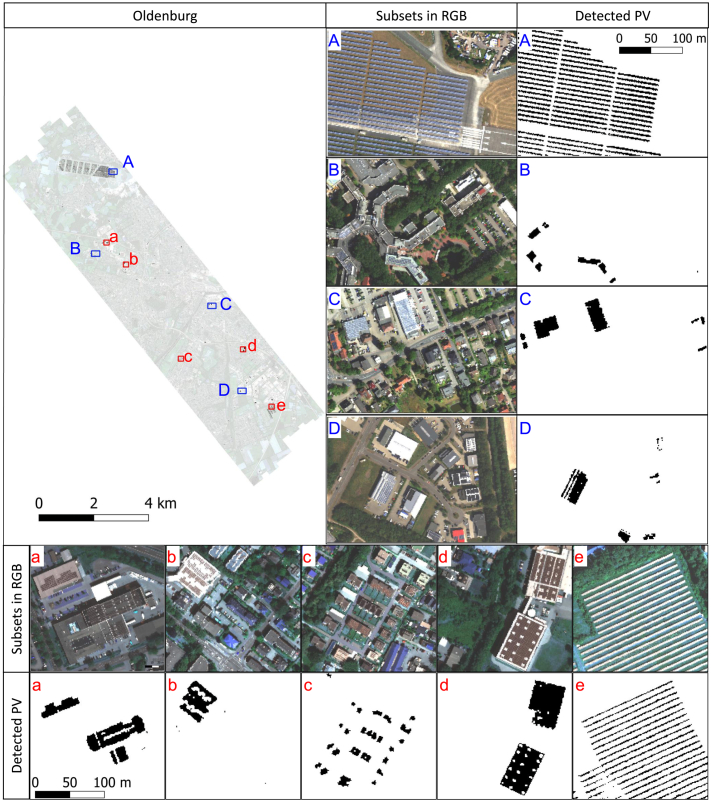
The overall and enlarged detection result in Oldenburg, as the detected PV areas were colored black. Four selected subsets (A, B, C, and D) were marked as blue rectangles in the overall map of Oldenburg, and the RGB and detected PV areas were enlarged on the right. For better illustration, five evenly distributed areas (a, b, c, d, e) were additionally marked in red in the overall map, and enlarged at the bottom.

The PV mapping results of the entire study area of Oldenburg were obtained. Fig. 10 shows the entire detected PV areas on the left side, about 170,000 pixels or 0.24 km^2^. On the right side four subsets were enlarged. In general, most PV modules were correctly detected within the four subsets. Either for the PV power plant of subset A, the campus roofs of subset B, the residential roofs of subset C, the industrial area of subset D, both locations and shapes were correctly detected. Further statistical results were obtained in the following validation process.

### 4.4 Validation

**Fig. 11:**
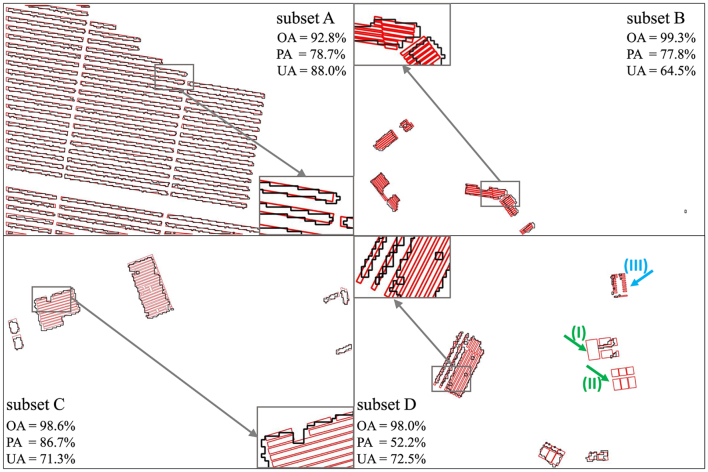
The PV mapping polygons compared with reference data in four subsets. Black polygons show the PV mapping areas, and red polygons show the reference data. The blue and green arrows in subset D show the omission error of the PV detection. To better illustrate the detection accuracy, a region for each subset was selected and depicted within the enlarged inlay figures. For each subset, Overall Accuracy (OA), Producer's Accuracy (PA) and User's Accuracy (UA) were acquired and presented.

To better compare the reference data and the detected PV areas, the OA, PA, and UA were acquired for four subsets. Together with magnified inlay areas, the validation results were presented in Fig. 11.

The Overall Accuracies (OAs) of the four subsets range between 92.8% and 99.3%, indicating that the methodology developed for this study works well. As shown in Fig. 11, each PV object was correctly detected except for several panels in subset D. Subset A covers many PV objects, and the detected PV polygons matched well with the reference data under the condition of 1.2 m spatial resolution of HySpex data. The same detection efficiency was shown in Subset C. Subset B has a PA of 77.8% and a UA of 64.5% since the displacement between the reference data and the HySpex data, as well as the distortion in the reference data from the 3K camera orthorectification. Subset D yielded a PA of 52.2% and a UA of 72.5%, mainly because areas (I) and (II) were omitted from classification as they are thin film PV modules rather than polycrystalline PV or monocrystalline PV. Furthermore, area (III) was partly missed because the PV modules were too small to be the prevailant spectra at 1.2 m resolution.

## 5 Discussion

### 5.1 PV spectral indices derivation

Our approach exploits physical absorption and reflectance features of PV modules. Two spectral features present in EVA film and C-Si in PV modules are particularly important for PV detection: The hydrocarbon absorption feature at 1.73 μm is very indicative for hydrocarbon-bearing materials. Kokaly et al. (2013) discussed the advantage of using the 1.73 μm instead of the 2.3 μm absorption feature in oiled material detection, which holds the same significance in PV detection. First, the 2.3 μm absorption feature can be confused with carbonate absorption in soil, but highly saline soils found in salt marshes are acidic with very low carbonate content. Second, the spectra of dry vegetation also exhibit the 2.3 μm absorption feature (Kokaly and Skidmore, 2015), which are primarily derived from structural biochemical constituents comprising plant cells. Therefore, using the hydrocarbon absorption feature at 1.73 μm as the primary spectral feature to detect PV is a better choice. The second feature is the steep increase in reflectance spectra of PV modules due to the rapid spectral increase of C-Si from 600 nm to 1150 nm (Schinke et al., 2015, Deng et al., 2017). The NSPI introduced by Czirjak (2017) addresses this feature. As Fig. 9 shows, implementing NSPI only is not sufficient to constrain most roofing tiles and a few copper roofs in PV detection. This would lead to confusion since most PV modules were installed on roofs with roofing tiles.

The present approach is able to deal with different detection angles and PV installation angles. The nHI, obtained by normalizing the existing HI, mitigates Bidirectional Reflectance Distribution Function (BRDF) effects from the laboratory spectra-goniometric measurements, and shows higher robustness in experimental results. Fig. 6 shows the variation of HI values among different detection angles, and nHI removed this variation. The nHI outperforms HI in dealing with material spectral variability and thus offers a better separation. Fig. 7 presents the statistical results of HI and nHI values with five materials. Both HI and nHI can easily distinguish two PV materials from two bitumen materials and a PVC sample. However, using HI values to distinguish PV from other materials leads to misclassifications. The nHI has higher separability between two PV materials and other materials. In addition, HI or nHI alone can barely distinguish between monocrystalline PV and polycrystalline PV. Fig. 8 shows that nHI has a better performance on the HyMap image spectral library in comparison to HI. HI failed in most hydrocarbon-bearing materials such as most roofing polyethylene, almost all polyethylene surface, and part of synthetic turf, because these materials all have the hydrocarbon absorption feature, and appropriate HI value range is difficult to define. Other than that, HI also failed in checking some spectra of roofing aluminum, roofing tiles, and synthetic turf. nHI only failed in some roofing polyethylene spectra and three pond spectra, leading to more robust results.

A combination of spectral indices is necessary in PV detection because the PV modules are composed of different materials. Using nHI only could cause confusion with hydrocarbon-bearing materials. Still, nHI is a good baseline, and other indices capture additional spectral features of PV or other similar polyethylene materials. Therefore, the combination of HI and other indices increases the detection accuracy by avoiding false positives. Fig. 9 shows that either nHI or the NSPI works well for most surface materials, but not all. Applying nHI alone would misclassify roofing polyethylene as PV modules, and applying NSPI only would misclassify roofing tiles as PV modules.

The approach was trained and assessed with different data types to parameterize the spectral indices, either with respect to different sensors (ASD, HyMap and HySpex) or different experimental conditions (laboratory spectra-goniometric measurements and airborne imaging spectroscopy data). The large HyMap imaging spectral library was employed to develop other spectral indices to constrain other materials for this study, including 31 surface material classes in 5627 labeled spectra. Therefore, the approach is considered robust to detect PV modules with imaging spectroscopy data from different sensors.

### 5.2 PV mapping results with airborne HySpex imaging spectroscopy data

The approach could quickly and efficiently detect C-Si based PV modules accurately. In general, Fig. 11 shows the OA of subset A, B, C and D exceed 90%. If we take a close look at the subsets, only a small section on the east side of subset B was incorrectly classified as a PV module. All other PV installations were correctly detected.

Specially, our approach is able to accurately detect PV modules in different arrangements and within different environments, without the need for explicit training samples for each setting, but purely based on their spectral characteristics. For example, each line of PV modules in the ground-based PV power plant was correctly and accurately detected (see subset A in Fig. 11). Had an object-segmentation based machine learning approach been employed instead, it would have been necessary to sample a sizeable number of labeled training data of PV installations in various arrangements of ground-based settings in addition to samples from residential and industrial roof tops. Hence, a clear advantage of the presented approach is the ability to perform well even in the absence of large sets of labeled training data.

The displacement between hyperspectral data and reference data as well as the distortion in 3K data are the main reason for a UA of 64.5% in subset B, while UAs of other subsets are relatively high. As shown in Fig. 11, the PV arrays in the magnified area of subset B were shifted from line arrangement, resulting in the low validation values. This is because the 3K camera is a framing system and therefore affected by relief displacements, which are only partially corrected in the ortho-rectification process. But since a digital elevation model was used, the height of objects above the Earth surface is not taken into account. Therefore, there are still relative geometric displacements that vary with respect to the building height relative to the Earth surface and additionally increases radially from the perspective center of the 3K image tiles.

Apart from the technical issue, the PA and UA values of four subsets show a limitation of the study. The low spatial resolution of HySpex data of 1.2 m and the resulting over- or under-classification at the edges of PV arrays is the main factor leading to the PA values in the validation. There is a distinct comparison of PA values between subset A and subset C. As can be seen in the enlarged inlay figure (Fig. 11), each PV module in subset A and subset C were detected. However, subset C has a PA of 86.7%, while subset A has a PA of 78.7%. This is because subset A contains more objects and therefore more edges were included. The area (III) in subset D also shows the detection limits due to the spatial resolution of 1.2 m of the HySpex data. Some small PV modules were ignored in this detection, since they were too small and scattered distributed, and therefore only covered by spectrally mixed pixels.

Furthermore, the thin film PV modules should be considered critically in the application of the approach. Although silicon solar cells dominate the market with a share of more than > 90%, and thin film PV modules have a small share due to low efficiency (Silvestre et al., 2018), it should be noted that thin film PV modules could not be detected by our approach. In subset D, area (I) and (II) were not identified (Fig. 11) because their spectra are very different from silicon PV modules. Thin film PV modules are made of different layers and exhibit non-specific characteristics in spectra. A clear identification and differentiation from other materials would require to measure it in the laboratory to explore their spectral reflectance characteristics. Since this PV module is quite rare, it was not available for this study.

In summary, the quality of the PV panel identification is very high (high OA). The lower PA and UA is mainly due to the low spatial resolution of the HySpex data as well as the geometric displacement between the validation and HySpex data.

### 5.3 Future directions

Although the robust approach could be transferred and applied to data collected by different sensors, the band selection of spectral indices would vary slightly. In particular, nHI and NSPI, as the dominant and most efficient indices for PV detection, are quite sensitive with their thresholds to the spectral bands of different sensors which they are applied to. Our study developed and combined these spectral indices for the first time, and trained their thresholds using laboratory spectra-goniometric and HyMap spectral libraries, then applied these thresholds with HySpex data on Oldenburg, and acquired accurate PV distributions. Therefore, it is demonstrated that these thresholds are relatively robust for sensors with different spectral resolutions. However, when the given data is only from a single sensor, adjusting the thresholds of these spectral indices, especially nHI and NSPI, could help to increase the detection accuracy. In particular, nHI and NSPI, as the dominant and most efficient indices for PV detection, are quite sensitive with their thresholds to the spectral bands of the different sensors to which they are applied. The future research could apply machine learning methods to define the thresholds for each spectral index based on training with massive pure spectra.

The physics-based approach presented in this study yields the potential to include new PV materials such as the new thin film PV modules that could not been detected, because samples were absent in the used spectral libraries. In a first step, these characteristics need to be investigated in a laboratory and tested in airborne or UAV-borne data to then derive robust spectral features that can be parameterized for a broader application. A large amount of training data across different regions is not necessary, if the spectral variability can be detected. This makes the physics-based approach a robust and practical method for PV detection.

Detecting large PV modules regionally or nationwide with spaceborne imaging spectroscopy data is efficient and useful in energy system modeling. Currently, the ongoing missions, such as the Italian PRISMA mission (Loizzo et al., 2019) and the upcoming German EnMAP mission (Guanter et al., 2015) are promising data sources for large area PV detection. However, since most spaceborne imaging spectroscopy data have a relatively coarse spatial resolution of 30 m × 30 m, PV mapping in urban areas could be challenging. In this context, a building mask would help improve the mapping accuracy when the detection target is PV systems on roofs. However, results show that the detection of large PV power plants outside urban areas works with high precision. Thus, future investigations could focus on monitoring such sites with spaceborne imaging spectroscopy data. Since effects such as PV soiling due to dust or pollen reduce the efficiency of PVs, this is of high importance to PV power plant operators and owners. Moreover, adding texture, shape, or other visual features from data with high spatial resolution is a great potential for future studies.

Future studies could explore to distinguish different types of PV based on their unique spectra such as the mono- and polycrystalline silicon, cadmium telluride (CdTe), copper indium selenium (CIS), and copper indium gallium selenide (CIGS) modules (Visa et al., 2016, Burduhos et al., 2018).

## 6 Conclusion

PV modules are not a pure material, but a combination of several materials. Therefore, their detection with imaging spectroscopy data should consider a combination of spectral indices that are able to separate PV modules and spectrally similar materials. The applicable and robust approach proposed in this study was validated on a large database (spectra-goniometric data, HyMap spectra library, and the HySpex imagery for Oldenburg), and yielded accurate PV areas. Furthermore, BRDF effects due to different detection angles in PV detection were observed and addressed by normalizing the hydrocarbon index. Moreover, the spatial resolution of the imaging spectroscopy data should be sufficient to detect PV material as pure pixels.

This study aims to create greater awareness of the potential importance of imaging spectroscopy data for PV identification. As a physics-based approach, it is robust, transferable, and can provide data on PV coverage on a regional or global scale in short time. The highlighted analysis of the normalized hydrocarbon index could tackle the detection angle problem in PV installations and data acquisition time, which evidently increases the PV detection accuracy.

It should be noted that the present approach was developed to detect the Si-based PV modules with EVA covers and needs to be further refined and updated to detect other PV modules. Future studies that employ spaceborne imaging spectroscopy data to detect large PV power plants modules should focus on monitoring in order to investigate the potential to detect soiling effects that can decrease the efficiency of such PV modules. The robustness of the developed and tested novel physics-based detection approach for PV power plants paves the way for more refined investigations towards PV type differentiation and the analysis of the efficiency of such modules.

## Author statement

W. Heldens and M. Schroedter-Homscheidt conceived the idea.

S. Weyand and M. Schroedter-Homscheidt organized the airborne campaign and collected validation data at Oldenburg test sites.

U. Heiden and A. Hueni organized the laboratory goniometer measurements, and assisted in the post-processing and interpretation.

W. Heldens and U. Heiden provided the HyMap spectral library.

C. Ji performed the algorithm development with support of W. Heldens and U. Heiden.

M. Bachmann and J. Zeidler support the technical parts of the study.

T. Lakes, H. Feilhauer, W. Heldens, U. Heiden, M. Schroedter-Homscheidt, M. Bachmann and T. Esch supervised the study.

S. Weyand, A. Metz-Marconcini and M. Schroedter-Homscheidt clarified the background of the study.

C. Ji and S. Weyand conducted the original draft preparation of the manuscript.

All authors contributed to the final review and editing of the manuscript.

## Funding

The study was supported by the e-shape project [European Union's Horizon 2020 research and innovation program, grant no. 820852], the China Scholarship Council [grant no. CSC 201806220088], and the project of “Development of a concept for information retrieval about the building stock in Germany with remote sensing” [German Federal Institute for Research on Building, Urban Affairs and Spatial Development within the Federal Office for Building and Regional Planning, grant no. 10.08.17.7-18.13].

## Declaration of Competing Interest

The authors declare that they have no known competing financial interests or personal relationships that could have appeared to influence the work reported in this paper.
